# The Dual Blockade in the Neoadjuvant Setting of HER-2 Positive Early-Stage Breast Cancer

**DOI:** 10.25122/jml-2019-0115

**Published:** 2019

**Authors:** Lucian Pop, Ioan Dumitru Suciu, Olivia Ionescu, Paris Ionescu, Oana Daniela Toader

**Affiliations:** 1. Department of Obstetrics and Gynecology, Institute of Mother and Child Care, Bucharest, Romania; 2. Department of General Surgery, Floreasca Emergency Hospital, Bucharest, Romania; 3. Faculty of General Medicine, Carol Davila University of Medicine and Pharmacy, Bucharest, Romania; 4. Department of Obstetrics and Gynecology, Nürnberg Hospital South, Nürnberg, Germany; 5. Department of Obstetrics and Gynecology, Ovidius University, Constanta, Romania

**Keywords:** Breast cancer, neoadjuvant, Trastuzumab, Pertuzumab, dual blockade

## Abstract

Patients with positive Her-2/neu breast cancer and a high risk of recurrence are known to benefit from the addition of the dual blockade of Her-2/neu with Trastuzumab and Pertuzumab to the neoadjuvant chemotherapy, a combination which has been demonstrated to give a higher rate of a complete pathologic response in the breast and in the axilla.

The purpose of this review is to outline the efficacy of the dual blockade with Trastuzumab and Pertuzumab in the neoadjuvant treatment of high-risk Her-2 positive breast cancer.

Electronic databases (Pubmed, Medline, and Cochrane Database of Systematic Reviews) were searched for English- and German-language studies, which were published in the last ten years. The search has been focused on neoadjuvant clinical trials as well as on the data presented in the abstracts published at the San Antonio Breast Cancer Symposium as well as at the annual meeting of the American Society of Clinical Oncology.

The results reported in the published clinical trials demonstrated a higher pathologic complete response rate in breast and lymph nodes after using targeted therapy with two anti-Her-2/neu agents - Trastuzumab and Pertuzumab in combination with neoadjuvant chemotherapy for early-stage Her-2/neu positive breast cancers. The pathologic complete response rate is the most important prognostic marker in Her-2/neu positive tumors, a higher pathologic complete response rate being demonstrated to be associated with a better survival outcome in terms of higher overall survival and disease-free survival rates.

## Introduction

Breast cancer (BC) is the most frequent type of cancer among genital malignancies and the second cause of death in women diagnosed with cancer [1]. Almost one-quarter of malignant breast tumors overexposes the protein Herceptin 2, which classifies them as Her-2 positive BCs [2]. This subtype of BC has an aggressive course associated with a high risk of recurrence and a worse clinical outcome [3]. In this case, neoadjuvant chemotherapy (NAC), in combination with a targeted agent against Her-2 neu Receptor, is associated with a high pathologic complete response (pCR) rate but controversial cardiac side effects [4]. In order to eradicate a therapy resistance to one monoclonal antibody, combination therapy of two anti-Her-2 neu agents results in better overall survival (OS) and disease-free survival (DFS) rates outcomes due to the synergistic effect of the dual blockade [5]. Moreover, the NAC in advanced BC is known to improve the success rate of surgery while adding a dual blockade to a NAC regimen has been shown to result in a higher pCR and, consequently, with better survival outcomes [6]. Currently, the dual blockade with the anti-Her-2 neu agents - Trastuzumab and Pertuzumab, together with a NAC regimen is the most effective with regard to the OS rate in the treatment of Her-2 neu positive BC [7,8]. Besides Trastuzumab and Pertuzumab, other anti-Her-2 neu agents have been tested in the neoadjuvant setting of BC, including T-DM1 and Neratinib, in order to select the combination therapy which better target the Her-2 Receptor [9,10]. In this article, we aimed to review the efficacy of the dual blockade with Trastuzumab and Pertuzumab in the neoadjuvant treatment of Her-2 neu positive BC with regards to the OS and DFS rates as outlined in clinical trials, as well as emphasizing the adverse effect of the dual blockade focusing on the cardiotoxicity.

### Dual blockade in the neoadjuvant setting

The combination of Trastuzumab and Pertuzumab with a NAC regimen in Her-2 neo positive BChas been tested in a phase II clinical trial - the NeoSphere trial in which 417 women with BC Her-2 new positive of stages II and III have received Trastuzumab, Pertuzumab with or without Docetaxel, being randomly included in four groups: Trastuzumab plus Docetaxel, Pertzumab plus Docetaxel, Trastuzumab and Pertuzumab plus Docetaxel and Trastuzumab and Pertuzumab without NAC. After the operation, the therapy has been continued with Fluorouracil, Epirubicin and Cyclophosphamide (FEC), and Trastuzumab in combination with Docetaxel for the group of patients who did not receive Docetaxel in the neoadjuvant setting [11-13]. The trial has shown that the dual blockade in combination with Docetaxel is associated with a better pCR rate and namely almost 46%, compared to the pCR rate obtained from the use of Docetaxel either with Transtuzumab or Pertuzumab (29% and 24%,respectively) [11,12] and from the use of the dual blockade without chemotherapy (PCR rate of almost 17%) [13]. The dual blockade and Docetaxel has also been associated with a 3-year progression-free survival (PFS) rate of 90% compared to the 3-year PFS of 86% of the women who received Docetaxel and Trastuzumab [11,13].

The TRYPHAENA trial obtained a pCR rate of 61.6% when using a regimen of six cycles of Docetaxel-Carboplatin-dual blockade compared with pCR rates of 57.3% and 66.2% when using three cycles of FEC followed by three cycles of Docetaxel and the dual blockade and three cycles of FEC with the dual blockade followed by three cycles of Docetaxel [11, 14].

The trial included women with both positive and negative hormonal status even though higher pCR rates have been observed for the breast tumors that did not express the hormonal receptors [14]. Furthermore, the absence of the hormonal receptors is considered to be associated with a higher pCR as women with negative hormone receptor status do respond better to the targeted therapy with the dual blockade [15]. A better response of the ER- Her-2 neu positive tumors to the targeted therapy seems to be based on the fact that ER- tumors are a subtype of the molecular Her-2 neu enriched tumors while ER+ tumors are classified as Luminal B tumors [16]. This has been outlined in a phase three clinical trial published in 2014 by Carey et al. who showed that Her-2 neu positive tumors biopsy samples that were obtained preoperatively are sensible to the targeted therapy compared to Luminal A and B tumors, in the latter two subtypes resulting a higher amount of residual disease.

The reported pCR rates for ER-, Her-2 neu positive tumors were 61.3 % and 41.3% in 2 phase three and two clinical trials, respectively [15,16] compared to 41.6% and 28.8% for the ER+, Her-2 neu positive tumors. The dual blockade, in combination with NAC, resulted in a better pCR rate compared with NAC with Transtuzumab alone and namely 77% compared to 55% [17].

Another biomarker that has been showed to predict the tumor response to the targeted therapy is a higher level of Her-2 neu receptor expression [18], a level that is typically detected using the Hermark assay [11]. A higher level of the Her-2 net protein in both ER+ and ER- tumors appeared to result in a high pCR rate as well as in a higher PFS rate after using the dual blockade in combination with a NAC regimen [18].

### Negative effects of the dual blockade

The most known adverse effect of a Her-2/neu monoclonal antibody is the effect on the cardiac function and namely the decrease of cardiac function. The combination of two anti-Her-2/neu agents would reduce cardiac function [19]. However, a combination therapy with FEC-dual blockade followed by adjuvant Docetaxel and dual blockade for one year resulted in a reduction of the left ventricular ejection fraction to less than 50% in only 7% of women while symptomatic systolic dysfunction of the left ventricle has been observed in only 4% of the patients [14,19]. The NeoSphere trial reported similar results with an insignificant increase in the incidence of left ventricular systolic dysfunction and left ventricular ejection fraction when using Trastuzumab and Pertuzumab with a NAC [12]. The reported percentages were 3% women who suffered a symptomatic left ventricular dysfunction after being subjected to Docetaxel and the dual blockade, compared with 1% of women who received Docetaxel and Pertuzumab, 1.1% women who received Docetaxel and Trastuzumab and no case of cardiac dysfunction in the group which received only the dual blockade [11,12]. Similarly to the low rates of systolic dysfunction and reduced left ventricular ejection fraction, a low incidence of cardiac failure class III and IV considering the classification of the New York Heart Association (NYHA) after using the dual blockade in combination with an anthracycline-based NAC regimen has also been reported. The reported rate of NYHA class III and IV cardiac failure was lower than 1% [20], while other clinical trials concluded that the dual blockade does not have any clinically significant adverse effects on the cardiac function [15,16].

Other commonly reported adverse effects of the dual blockade are febrile neutropenia, leukopenia, alopecia, diarrhea and nausea, all of these side effects being also frequently encountered when using a chemotherapeutic schema both in the adjuvant and in the neoadjuvant setting. A high incidence of febrile neutropenia of almost 36% has been observed when using Trastuzumab and Pertuzumab with anthracycline-based NAC [11].

## Conclusion

All the clinical trials which have been mentioned in this review have demonstrated a clinical benefit of using the dual blockade with Trastuzumab and Pertuzumab in combination with a neoadjuvant chemotherapy regimen for women with early-stage Her-2 neu positive breast cancer. The clinical benefit for these breast tumors is expressed as the achievement of a higher pCR rate after using the dual blockade in the neoadjuvant setting. The synergistic effect of two monoclonal anti-Her-2/neu agents has been demonstrated to be associated with an increase of the pCR with almost 18% compared to the pCR rate resulted after using only one anti-Her-2/neu agent independently of the NAC regimen. Moreover, studies have shown that breast tumors with poor prognosis and, namely with negative hormonal receptor status and positive Her-2/neu receptor, are sensitive to the dual blockade compared with breast tumors generally associated with better prognosis (ER+).

When it comes to the adverse effects, the dual blockade has not been demonstrated to increase rates of systolic dysfunction or heart failure NYHA class III and IV compared to Transtuzumab or Pertuzumab alone with NAC.

## Conflict of Interest

The authors confirm that there are no conflicts of interest.
